# Limiting the effects of radiation damage in MicroED through dose selection during data processing

**DOI:** 10.1107/S205979832500912X

**Published:** 2025-11-13

**Authors:** Henri Colyn Bwanika, Jingjing Zhao, Gerhard Hofer, Uwe H. Sauer, Hongyi Xu

**Affiliations:** a Department of Material and Environmental Chemistry Stockholm University Stockholm Sweden; b Department of Chemistry Umeå University Umeå Sweden; c Research School of Chemistry Australian National University Canberra Australia; Institut de Biologie Structurale, France

**Keywords:** microcrystal electron diffraction, MicroED, data processing, macromolecular structure, radiation damage

## Abstract

Microcrystal electron diffraction (MicroED), also known as three-dimensional electron diffraction (3D ED), allows the collection of diffraction data from submicrometre-sized crystals under low electron-dose conditions. Despite having several advantages over conventional X-ray crystallographic techniques, susceptibility to radiation damage is a great challenge that remains to be solved in MicroED. Similar to X-ray crystallography, radiation damage to the macromolecular crystal structures in MicroED manifests in two forms: global damage that affects the overall order of the crystal lattice and site-specific damage that affects highly sensitive residues and moieties in macromolecules. Traditionally, the unit e^−^ Å^−2^ has been used for electron-dose estimations, which does not consider the interaction between the incident electron beam and the sample. In this study, we clarify the terminology for describing ‘dose’ in electron crystallography, including the procedure for converting values from e^−^ Å^−2^ to grays (Gy). Furthermore, we investigated data-processing strategies that could be used to limit the effects of radiation damage to the crystal. During MicroED data collection, radiation damage increases with the number of acquired ED frames because the accumulated electron dose increases. Data collected from several crystals and processed in this way can be merged to increase the completeness and subsequently be used for structure refinement. According to our results, this approach improves the resolution of the data, the data statistics, the structure determination and the quality of the final structure.

## Abstract 2

The effect of radiation damage in MicroED has been studied. By only including data collected at low accumulated electron dose during data processing, the effects of radiation damage can be reduced.

## 1. Introduction

X-ray diffraction (XRD) has been an indispensable tool in structural biology and structural chemistry since the early 1960s, when the first crystallographic structures of myoglobin (Kendrew *et al.*, 1960 ▸) and hemoglobin (Perutz *et al.*, 1960 ▸) were determined. As of September 2025, the Protein Data Bank (PDB) holds more than 240 000 entries (Berman *et al.*, 2000 ▸), of which 85% are crystal structures, which provide a basis for understanding biological processes and diseases and for structure-guided drug discovery. However, the growth of perfect, diffraction-quality protein crystals is difficult to control, and problems such as crystal twinning and pseudosymmetry can arise, making structure determination difficult (Rupp, 2010 ▸). Moreover, during data collection, radiation damage to the crystal by the illumination medium, *i.e.*X-rays, or electrons immensely affects the data accuracy and final structure determination (Henderson, 1995 ▸; Berglund *et al.*, 2002 ▸; Garman & Owen, 2006 ▸; Liebschner *et al.*, 2015 ▸; Garman & Weik, 2017 ▸, 2023 ▸; Hattne *et al.*, 2018 ▸).

Radiation damage is common in XRD, where photoionization, due to high rates of photoelectric absorption from X-ray interaction with matter, knocks out inner-shell electrons from the atoms (Nass, 2019 ▸), leading to highly mobile secondary electrons that cause thermal diffusion and unstable molecular radicals (Jones *et al.*, 1987 ▸; Nass, 2019 ▸). The damage caused by secondary electrons can be further amplified through Auger electrons, which result in further ionization events and disorder to the crystal lattice during data collection (Jeng & Chiu, 1984 ▸). While thermal diffusion of radicals can be effectively reduced by vitrification of the crystals and data collection at 100 K (Jeng & Chiu, 1984 ▸; Garman & Owen, 2006 ▸), the radiation-damage effects of secondary electrons and Auger electrons resulting from high levels of photoelectric absorption events remain a major cause of damage in XRD. The unit for describing absorbed radiation dose of X-rays is the gray (Gy), defined as the absorption of one joule of radiation energy per kilogram of matter. To mitigate radiation damage in single-crystal XRD, sufficient diffracting material is required for the collection of data prior to destruction.

MicroED (Shi *et al.*, 2013 ▸; Nannenga & Gonen, 2016 ▸), also known as 3D ED (Gemmi *et al.*, 2019 ▸), is a promising method for the structure determination of biomolecules from sub­micrometre-sized crystals (Nannenga & Gonen, 2019 ▸; Clabbers & Xu, 2020 ▸, 2021 ▸). Currently, the most widespread method for MicroED data collection is continuous-rotation electron diffraction (cRED), in which the crystal is continuously rotated in the electron beam while electron diffraction patterns are collected in ‘movie’ mode on a fast read-out detector (Nederlof *et al.*, 2013 ▸; Wang *et al.*, 2017 ▸, 2018 ▸). The low electron-dose setup, cryogenic specimen holder and fast data-collection strategy allow researchers to study the crystal structures of a large variety of samples, such as zeolites (Huang, Willhammar *et al.*, 2021 ▸), metal–organic frameworks (MOFs), covalent organic frameworks (Huang, Grape *et al.*, 2021 ▸), small organic molecules, pharmaceuticals (Gruene *et al.*, 2018 ▸; Jones *et al.*, 2018 ▸) and proteins (Shi *et al.*, 2013 ▸; Xu *et al.*, 2019 ▸).

MicroED is complementary to existing methods in structural chemistry/biology and in particular to X-ray crystallo­graphy, where growing perfectly packed crystals of sufficient size is often a major hurdle inhibiting efficient structure determination. In MicroED, crystals too small to yield structures in XRD can be used, potentially bypassing time-consuming crystallization optimization. Despite this inherent advantage and recent advancements in MicroED and cryo-EM, electron beam-induced damage is still one of the predominant limiting factors in macromolecular structure determination (Hattne *et al.*, 2018 ▸; Henderson, 1995 ▸).

In contrast to X-ray crystallography, the most commonly used unit for describing ‘electron dose’ in electron microscopy is e^−^ Å^−2^, which is an estimation of the number of electrons illuminated on a unit area. However, e^−^ Å^−2^ more accurately corresponds to fluence rather than electron dose, and thus should not be used as a direct measure of dose. In Table 1 ▸, we summarize the correct terms that can be used for quantifying electron dose in electron diffraction experiments.

For macromolecular crystals, which mostly consist of C, N, O, S and H, elemental composition does not make a great difference when estimating electron dose by stopping power (Egerton, 2021 ▸). Instead, the electron dose is affected strongly by the acceleration voltage and exposure time. For pure water, based on stopping power-to-dose conversions (Berger *et al.*, 1999 ▸), 100, 200 and 300 keV electrons deposit 6.6, 4.5 and 3.8 MGy per e^−^ Å^−2^, respectively (Henderson, 1990 ▸; Zeldin *et al.*, 2013 ▸; Bury *et al.*, 2018 ▸). The typical fluence applied for cryo-EM single-particle analysis is around 40–50 e^−^ Å^−2^ at 300 keV, which is an electron dose of approximately 152–190 MGy (Hayward & Glaeser, 1979 ▸; Baker *et al.*, 2010 ▸; Karuppasamy *et al.*, 2011 ▸; Grant & Grigorieff, 2015 ▸). Meanwhile, the common electron-dose threshold for continuous-rotation MicroED experiments is approximately 23 MGy (Nannenga *et al.*, 2014 ▸).

The accumulation of global damage can be detected as a loss of crystal lattice order as well as a loss of high-resolution data in the diffraction pattern (Hattne *et al.*, 2018 ▸; Weik *et al.*, 2000 ▸). In addition, site-specific damage has been documented to affect certain moieties which are more susceptible to radiation damage than others (Meents *et al.*, 2010 ▸; Petrova *et al.*, 2010 ▸; Gerstel *et al.*, 2015 ▸). Such local changes have been observed in XRD in dose-fractionation studies, which show an incremental reduction of disulfide bridges (Weik *et al.*, 2000 ▸) and loss of signal from carboxylate moieties (Burmeister, 2000 ▸). This site-specific damage around disulfide bonds and carboxylic side chains has been shown to occur at doses of as low as 11.6 MGy (a fluence of 2.59 e^−^ Å^−2^) in MicroED (Hattne *et al.*, 2018 ▸). Although collecting MicroED data with low electron flux density (Hattne *et al.*, 2018 ▸) is promising, it may be difficult to obtain a sufficient signal-to-noise ratio using a scintillator-based or hybrid pixel detector, as we have demonstrated in Supplementary Fig. S1. The number of electrons cannot be arbitrarily decreased, because there must still be sufficient signal to confidently analyse each data set. Therefore, it is of high importance to analyse how electron beam-induced damage affects the quality of 3D ED/MicroED data and understand how data could be treated and processed to improve structural analyses.

Here, using microcrystals of hen egg-white lysozyme (HEWL) and human carbon anhydrase isoform II bound with acetazolamide (HCA II:AZM), we report a strategy that can be applied during data processing to mitigate the effects of radiation damage to the crystal. The reported data-processing strategy involves utilizing only the few first frames of data sets collected from a large number of randomly oriented crystals, and our results show improved data-processing statistics and high-quality final structure models. The processing strategy is based on the assumption that the data were collected from crystals randomly orientated on the cryo-EM grid and have a large overall angular coverage. Through this work, we also encourage the electron crystallography community to use the correct terminology when describing electron ‘dose’.

## 2. Experimental methods

### 2.1. Crystal growth

#### 2.1.1. Tetragonal lysozyme crystals (lysozyme:native)

Tetragonal hen egg-white lysozyme microcrystals were grown as described previously (Falkner *et al.*, 2005 ▸; Barends *et al.*, 2014 ▸), but skipping the cross-linking step. They were stored in 8%(*w*/*v*) NaCl, 0.1 *M* sodium acetate buffer pH 4.0.

#### 2.1.2. Tetragonal lysozyme crystals soaked with GdCl_3_ (lysozyme:GdCl_3_)

The native hen egg-white lysozyme crystals were soaked with 100 m*M* GdCl_3_ and incubated at room temperature for at least 30 min before MicroED specimen preparation.

#### 2.1.3. Tetragonal lysozyme crystals soaked with Gd cluster (lysozyme:Gd)

The native hen egg-white lysozyme crystals were soaked in 100 m*M* gadoteridol [Gd^3+^:10-(2-hydroxypropyl)-1,4,7,10- tetraazacyclododecane-1,4,7-triacetic acid] (Girard *et al.*, 2002 ▸; Barends *et al.*, 2014 ▸) and incubated at room temperature for at least 30 min before MicroED specimen preparation.

#### 2.1.4. Tetragonal lysozyme crystals soaked with C_8_H_4_I_3_NO_4_ (lyoszyme:Na_I_3_C)

The native hen egg-white lysozyme crystals were soaked with 200 m*M* sodium 5-amino-2,4,6-triiodoisophthalic acid (sodium I3C, the magic triangle; Beck *et al.*, 2008 ▸) and incubated at room temperature for at least 30 min before MicroED specimen preparation.

#### 2.1.5. Human carbonic anhydrase isoform II (HCA II)

Crystals of HCA II were grown as described previously (Clabbers *et al.*, 2020 ▸) in sitting-drop vapour-diffusion setups using microbridges (Hampton Research) and 24-well Linbro crystallization plates (Hampton Research). The drops were prepared by mixing 10 µl protein solution (20 mg ml^−1^) with 10 µl precipitant solution (2.8 *M* ammonium sulfate, 0.1 *M* Tris pH 8.5) and were equilibrated against 1 ml reservoir solution consisting of the precipitant. Plates were incubated at 20°C and crystals appeared in 2–3 days. Crystals were harvested by crushing and repeated pipetting into 1.5 ml Eppendorf tubes to collect a slurry of small crystals in mother liquor and were stored at 20°C until cryo-grid preparation.

#### 2.1.6. Complex of HCA II with the inhibitor acetazolamide (HCA II:AZM)

The complex was prepared by soaking the crystal slurry with inhibitor solubilized in dimethyl sulfoxide (DMSO) for 20 min, with a final concentration of 0.5 m*M* HCA II and 4.5 m*M* AZM.

### 2.2. Sample preparation

All MicroED specimens were prepared using the Preassis method (Zhao *et al.*, 2021 ▸) with 37.2 mbar pressure. Lacey carbon grids with 3D nylon support (Wennmacher *et al.*, 2019 ▸), as well as Quantifoil R 2/1 grids were used in the case of lysozyme:native. Quantifoil R 1.2/1.3 grids were used in the lysozyme:native, lysozyme:Gd, lysozyme:GdCl_3_, lysozyme:Na_I_3_C, HCA II and HCA II:AZM cases. Before specimen preparation, the EM grids were glow-discharged with 20 mA current for 60 s using a PELCO easiGlow 9100 discharge system. For each individual sample, a 3 µl drop of protein suspension was deposited onto the EM grid. After the excess liquid had been removed, the grid was immediately plunged into liquid ethane for vitrification. For the detailed specimen-preparation protocol, please refer to Zhao *et al.* (2021 ▸).

### 2.3. MicroED data acquisition

MicroED data for lysozyme:native, lysozyme:Na_I_3_C, lysozyme:Gd, HCA II and HCA II:AZM were collected on a Jeol JEM-2100 transmission electron microscope with a LaB_6_ filament operated at 200 kV, equipped with a hybrid electron detector (Amsterdam Scientific Instruments, Timepix). Data collection was controlled by the *Instamatic* Python-based electron diffraction data-collection software (Cichocka *et al.*, 2018 ▸), with 1.5 s exposure and a rotation speed of 0.46° s^−1^. The flux density applied for data collection was 0.10 e^−^ Å^−2^ s^−1^, which results in an equivalent dose rate of approximately 0.45 MGy s^−1^.

Conversion between fluence and grays was performed using the equation
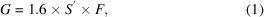
where *G* is the dose (in MGy), *F* is the fluence (in e^−^ Å^−2^) and *S*′ is the stopping power (in MeV cm^2^ g^−1^). The stopping power of protein crystals has been estimated to be 3.93, 2.69 and 2.24 MeV cm^2^ g^−1^ for 100, 200 and 300 keV electrons, respectively (Berger *et al.*, 1999 ▸; Egerton, 2021 ▸).

MicroED data for lysozyme:GdCl_3_ were collected on a Themis Z transmission electron microscope to study the effects of radiation damage on the metal-binding site. The microscope was operated at 300 kV and data were collected using a Gatan OneView camera. Data collection was controlled by a *DigitalMicrograph* script, *InsteaDMatic* (Roslova *et al.*, 2020 ▸), with 2 s exposure and 0.5° s^−1^ rotation speed. The flux density applied was 0.09 e^−^ Å^−2^ s^−1^, which is equivalent to 0.34 MGy s^−1^. We note that the eucentric height of the TEM stage was carefully calibrated before MicroED data were collected from each crystal. This is to ensure that the crystal remains in the electron beam throughout the continuous rotation (30–50°).

### 2.4. Data processing, including reduction and scaling

Data processing was performed using the *XDS* software package (Kabsch, 2010*a* ▸,*b* ▸). First, data sets from well diffracting crystals were screened for and ED frames were visually analysed with the *XDS* graphical user interface (Kursula, 2004 ▸). Initially, data collected from the best diffracting crystals were used for indexing and integration in space group *P*1 with all frames included (19 crystals of lysozyme:native, 14 crystals of lysozyme:Gd, 15 crystals of lysozyme:Na_I_3_C, nine crystals of lysozyme:GdCl_3_, 13 crystals of HCA and 20 crystals of HCA II:AZM). This is to ensure that the tilt range of individual data sets is sufficient for successful indexing. Next, to obtain data sets for different accumulated electron doses (frames), an extra iteration of processing with only the CORRECT step (JOB=CORRECT) was performed, during which the original processed data set was reindexed in the correct space group and the number of frames to be included in each data set was specified in the XDS.INP file.

MicroED data for lysozyme:native and lysozyme:GdCl_3_ were indexed and integrated in space group *P*4_3_2_1_2. MicroED data for HCA II:AZM were indexed and integrated in space group *P*2_1_. The following notation is used to refer to the frame intervals and corresponding electron doses: the first ten frames (1-10F), the first 20 frames (1-20F), frame 21 to frame 35 (21-35F) and all frames (All F). The respective calculated electron doses are summarized in Table 2 ▸. Following data processing, the obtained data sets were scaled and merged with *XSCALE* (Kabsch, 2010*b*).

### 2.5. Structure determination

The crystal structures of lysozyme:native and lysozyme:GdCl_3_ were solved with *Phenix* (Liebschner *et al.*, 2019 ▸) using *Phaser* and the automated molecular-replacement tool *Phaser-MR* (McCoy *et al.*, 2007 ▸) with the 1.33 Å resolution X-ray structure of tetragonal lysozyme, PDB entry 193l (Vaney *et al.*, 1996 ▸), as the starting model. The structure of lysozyme:GdCl_3_ was determined by molecular replacement using the *MOLREP* tool from the *CCP*4 suite (Agirre *et al.*, 2023 ▸) with PDB entry 4n5r (Barends *et al.*, 2014 ▸). The HCA II:AZM structure was solved by molecular replacement using the recently published MicroED structure of HCA II:AZM (PDB entry 6yma; Clabbers *et al.*, 2020 ▸).

### 2.6. Structure refinement

The crystal structures were refined with the *phenix.refine* tool (Afonine *et al.*, 2012 ▸) using atomic scattering factors for electrons. In the refinement, the following options were selected in the refinement strategy: *XYZ* (reciprocal space), rigid body and individual atomic *B* factors for each atom in a refined structure. The atomic *B* factors for each atom of the starting models in the first round of refinement were manually set to 20 Å. Only one round of refinement was performed and each round consisted of three iterations. Reference-model restraints (PDB entry 2yvb) were applied for the structure refinement of lysozyme:GdCl_3_. The test set used to generate *R*_free_ values consisted of 5% of the total reflections for the refinements in Tables 3 ▸ and 4 ▸ and 10% for the refinement in Table 5 ▸. Solvent molecules were automatically placed in the HCA II:AZM final models with resolution higher than 2.5 Å.

### 2.7. Model validation

The refined structures were inspected and modified against the calculated electron density in *Coot* (Emsley & Cowtan, 2004 ▸). Validation of the models was carried out using *MolProbity* (Chen *et al.*, 2010 ▸).

### 2.8. Graphics rendering

Figures of final structure and map densities (Figs. 4, 5, 6, 7 and Supplementary Fig. S4) were prepared and generated in *PyMOL* version 2.4.0 (Schrödinger). Supplementary Fig. S3 was generated using *Coot*.

## 3. Results

### 3.1. Effect of accumulated electron dose on global radiation damage

**Figure 1:**
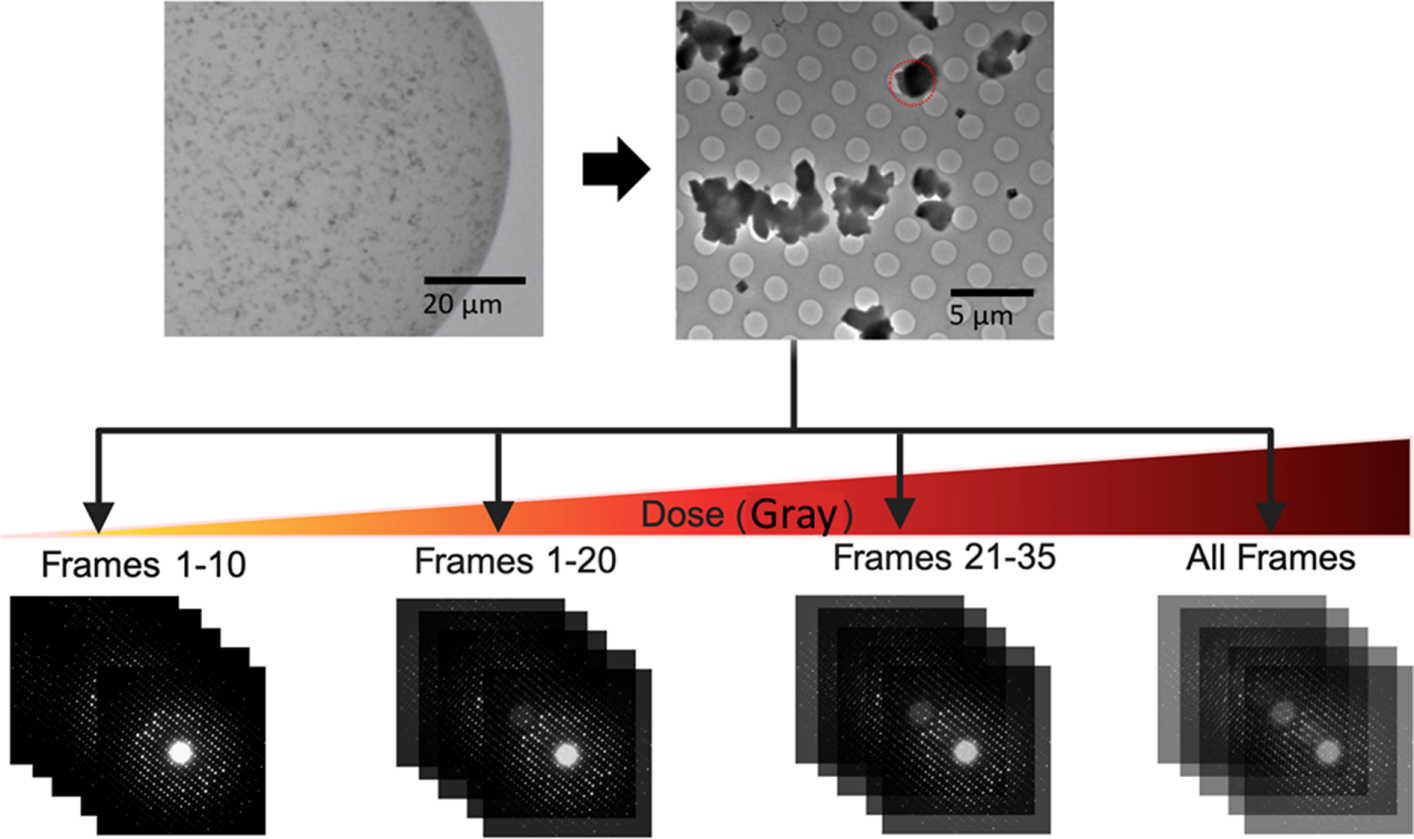
The data-processing strategy that was used to generate data sets with different frames and electron doses in this study. The top panel shows an optical microscope image of the crystallization drop and a low-magnification TEM image of the crystals (lysozyme:Gd in this case). The red circle shows a typical example of the area over which MicroED data are collected.

**Figure 2:**
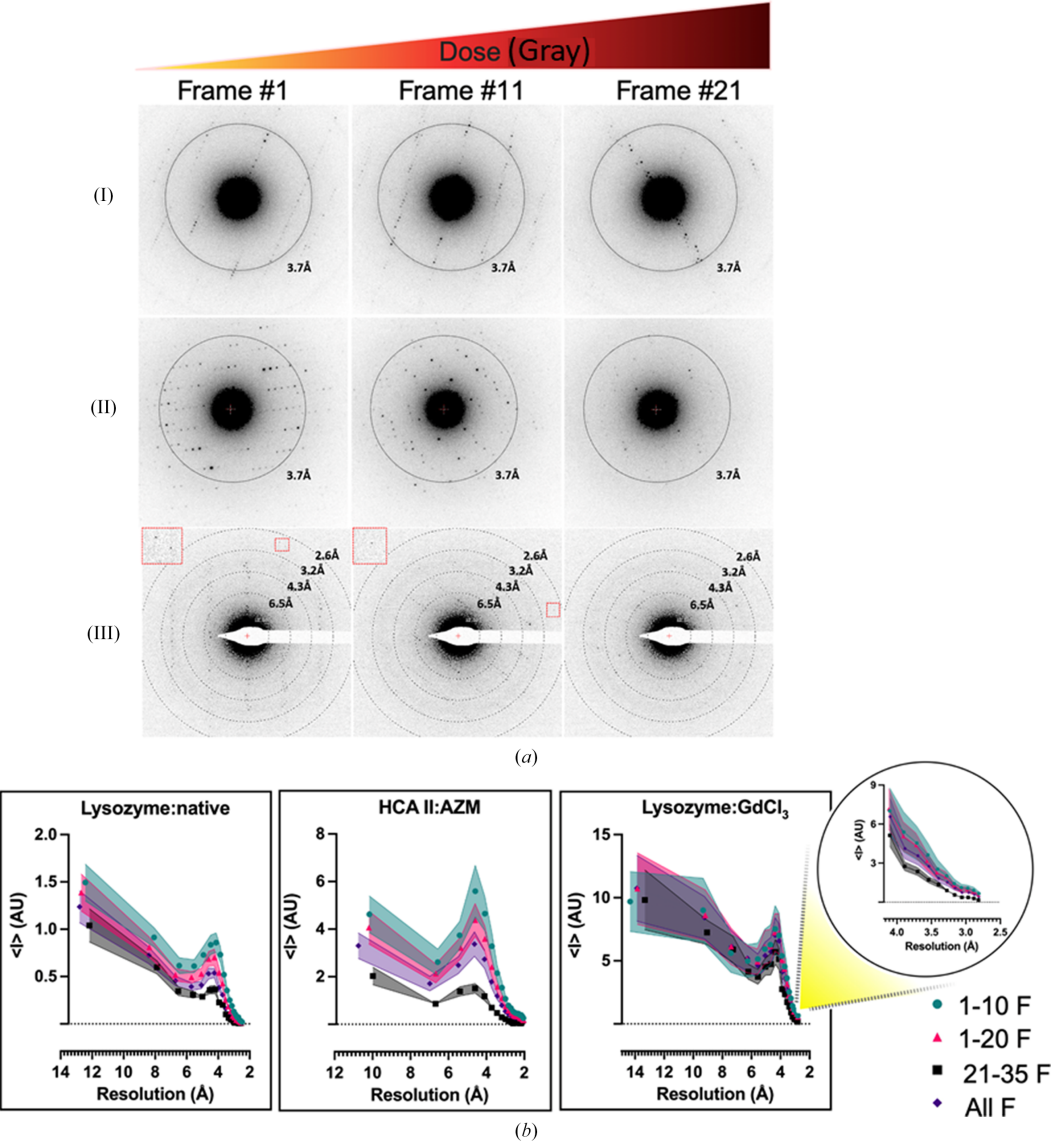
Effect of electron dose on the strength of integrated and unmerged mean reflection intensities 〈*I*〉. The flux density is approximately 0.10 e^−^ Å^−2^ s^−1^ (dose rate 0.45 MGy s^−1^ at 200 keV and 0.38 MGy s^−1^at 300 keV) and the exposure time is 1.5 s per frame. (*a*) Loss of diffraction-spot strength/intensity and resolution due to radiation damage with increasing frame number. Panels (I) and (II) show the trend of intensity decay in lysozyme:native and HCA II:AZM MicroED data, respectively, collected on a Timepix detector installed on a Jeol JEM-2100 microscope operated at 200 kV. Panel (III) shows diffraction patterns of lysozyme:GdCl_3_ collected on a OneView Themis Z microscope operated at 300 kV. (*b*) Intensity of diffraction spots as a function of resolution for the four data sets. 1-10F, first ten frames; 1-20F, first 20 frames; 21-35F, frame range 21–35; All F, all data. Overall, the 1-10F data set yielded reflections with higher mean intensities compared with other data sets. Data are plotted as 〈*I*〉 = mean *I*_*hkl*_ ± standard error of the mean versus resolution for a representative 24 crystals (lysozyme:native), 16 crystals (HCA II:AZM) and nine crystals (lysozyme:GdCl_3_). Note that in each case the same crystals are compared for the different doses. Lysozyme:native refers to native tetragonal lysozyme crystals deposited on a holey carbon grid with nylon support, HCA II:AZM refers to HCA II bound to its inhibitor acetazolamide and lastly lysozyme:GdCl_3_ refers to lysozyme soaked in GdCl_3_.

Using the data-collection strategy shown in Fig. 1 ▸, the loss of diffraction intensities due to global radiation damage accumulated in ED frames towards the end of data collection is shown in Fig. 2 ▸(*a*). Panels (I) and (II) show the trend of intensity decay in the lysozyme:native and HCA II:AZM MicroED data, respectively. These data sets were collected on a Jeol JEM-2100 microscope operated at 200 kV. Panel (III) shows diffraction patterns of lysozyme:GdCl_3_ collected on a ThermoFisher Themis Z microscope operated at 300 kV. Consequently, in Fig. 2 ▸(*b*) and Supplementary Fig. S2, the mean reflection intensities (〈*I*_*hkl*_〉) calculated by *XDS* were higher when frames 1–10 (1-10F) and frames 1–20 (1-20F) of diffraction data were used in data processing, compared with those when frames 21–35 (21-35F) and all data frames (All F) were used. For example, for 1-10F (6.75 MGy) and 1-20F (13.5 MGy) the mean reflection intensities were higher, especially in the case of lysozyme:native, lysozyme:Na_I_3_C, lysozyme:Gd, HCA II and HCA II:AZM, compared with 21-35F (total absorbed dose 23.63 MGy) and All F. As expected, using the 21-35F data set resulted in the lowest mean intensities, implying that later frames of a data set incurred a high level of radiation damage and should be omitted during data processing in order to optimize the quality of the data as well as the final charge-density maps. We note that in the lysozyme:GdCl_3_ case, which was collected on a 300 kV TEM, the mean intensities of the MicroED data were similar for frames 1–10, frames 1–20 and all frames in resolution bins lower than 3 Å. This suggests that beam damage can be reduced at comparable electron flux densities (e^−^ Å^−2^ s^−1^) when higher accelerating voltages, such as 300 kV, are applied, because the probability of inelastic scattering is lower at higher electron energies (Peet *et al.*, 2019 ▸). Nevertheless, a similar trend can be observed in higher resolution bins (highlighted on the far right in Fig. 2 ▸), where it becomes clear that high electron-dose data sets (21-35F and All F) resulted in lower intensities compared with the low electron-dose data sets 1-10F and 1-20F. Furthermore, it is clear that HCA II:AZM crystals were more sensitive to electron beam damage.

**Figure 3:**
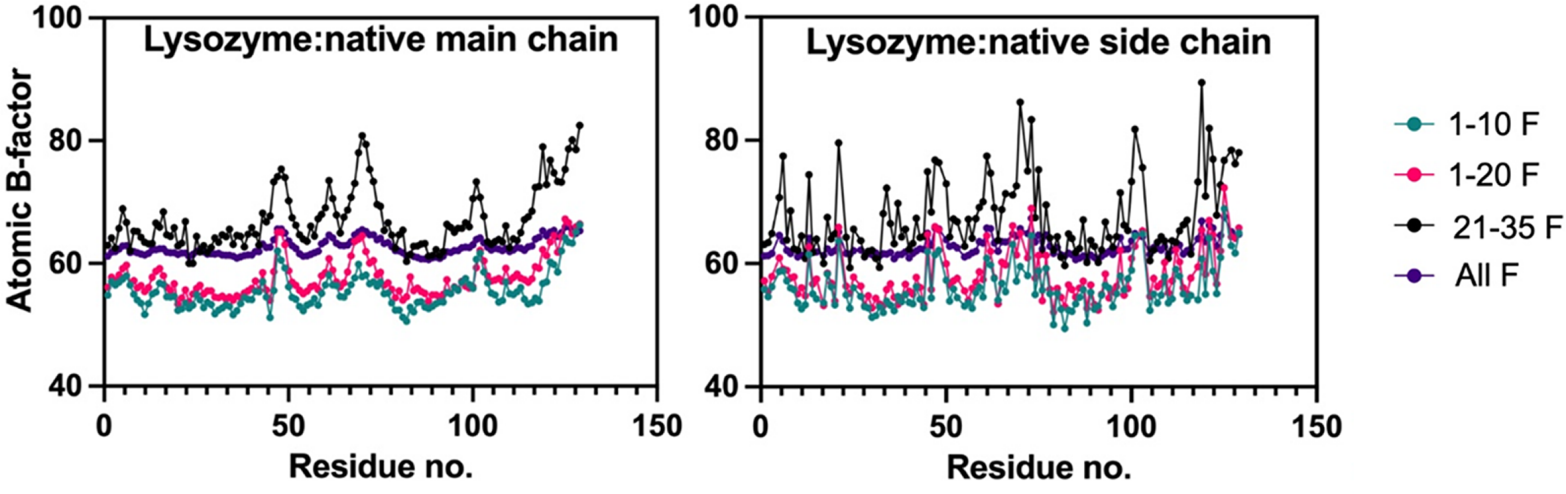
The effect of electron dose on atomic displacement factors. Atomic *B* factors were computed with *phenix.refine* (Liebschner *et al.*, 2019 ▸). Isotropic *B* factors of lysozyme:native for main chain and side chains are as indicated in the figure.

To further study the effect of the data-processing strategy on global radiation damage, we investigated how the atomic displacement parameters are affected during data processing. Wilson *B* factors, although unreliable indicators of radiation damage at low resolution (Rupp, 2010 ▸), tended to increase with increasing electron dose (Table 3 ▸). There was a strong correlation between an increase in electron dose and an increase in the atomic *B* factors of main-chain and side-chain atoms in both the lysozyme:native structure and the HCA II:AZM structure (Fig. 3 ▸). Moreover, an increase in atomic *B* factors of the AZM ligand and the water molecules (solvent) could be observed in the HCA II:AZM complex (Table 4 ▸).

### 3.2. Assessment of the data quality of the merged and scaled intensities

There are a number of parameters that can be monitored to verify the quality of the processed data. One such quality factor is *R*_meas_, which represents internal data consistency (Diederichs & Karplus, 1997 ▸). Measuring the mean signal-to-noise ratio *I*/σ(*I*) is another alternative to estimate data significance, as is the correlation coefficient (CC_1/2_), which is an ideal measure for assessing the best resolution cutoff of a data set (Evans, 2006 ▸; Evans & Murshudov, 2013 ▸; Karplus & Diederichs, 2012 ▸). The *R*_meas_ values used to evaluate internal data consistency are calculated using the equation
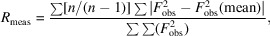
where *n* is the number of observations of a particular reflection. *R*_meas_ includes a correction for redundancy, making it more appropriate for comparing data sets with different multiplicities (Diederichs & Karplus, 1997 ▸).

There was an increase in the *R*_meas_ when we compared the low electron-dose data sets (1-10F and 1-20F) with the high electron-dose data sets (21-35F and All F), both for lysozyme:native and HCA II:AZM data. The *R*_meas_ for lysozyme:native increased from 0.566 (2.695) for 1-10F to 0.687 (5.063) for 1-20F and to 1.038 (16.610) for 21-35F and decreased to 0.844 (8.263) for All F (Table 3 ▸ and Supplementary Table S2). *R*_meas_ for HCA II:AZM increased from 0.352 (1.215) for 1-10F to 0.486 (1.804) for 1-20F and to 0.590 (3.173) for 21-35F and decreased slightly to 0.579 (2.637) for All F (Table 4 ▸ and Supplementary Table S4). The extremely high *R*_meas_ value reported for the 21-35F data set is an indication of how high electron doses affect the internal consistency of the data, especially in the high-resolution shells.

Based on the values of averaged *I*/σ(*I*) and CC_1/2_, the 1-20F and All F data-processing schemes are more desirable in all three cases, namely lysozyme:native, lysozyme:GdCl_3_ and HCA II:AZM (Tables 3 ▸ and 4 ▸). One of the noticeable downsides of omitting data as a strategy to mitigate radiation damage is that it significantly negatively affects the data completeness. This can be overcome by optimizing the data collection to obtain low-dose data sets with complete data wedges as well as merging a larger number of crystals.

### 3.3. The effects of using a limited number of frames on phasing and refinement quality

Following data processing, the final scaled and merged intensities were used to obtain the electrostatic potential maps and the initial structure models using molecular replacement by *Phaser-MR* (McCoy *et al.*, 2007 ▸) in *Phenix* (Liebschner *et al.*, 2019 ▸). Detailed accounts of refinement for respective electron doses are shown in Table 3 ▸ for lysozyme:native and in Table 4 ▸ for HCA II:AZM. Space group *P*4_3_2_1_2 was found for all determined structures of lysozyme crystals and *P*2_1_ in the case of HCA II:AZM.

As reported previously for the effects of global damage, the Wilson *B* factors generally increased with accumulated electron dose from 1-10F to 21-35F. We notice a slight decrease in Wilson *B* factors between the 21-35F and All F data sets. As with the *R*_meas_ values, this observation is reasonable since the 21-35F data sets mostly comprise of only the tail part of the data frames collected from crystals. Interestingly, the amount of discernible detail in the final structures improved with less accumulated dose. For example, the numbers of water molecules in the HCA II:AZM structures, as reported in Table 3 ▸, were 34 for 1-10F and 47 for 1-20F, compared with 31 for All F. These results suggest that it could be beneficial to omit data in order to avoid data frames in which the crystal has accumulated strong radiation damage. The ideal number of frames to be included in all three cases is 20, with an accumulated electron dose of 13.5 MGy (fluence of 3.0 e^−^ Å−^2^) in lysozyme:native and HCA II:AZM data collected at 200 kV and an accumulated electron dose of 13.6 MGy (fluence of 3.6 e^−^ Å^−2^) in lysozyme:GdCl_3_ data collected at 300 kV.

### 3.4. Effects of accumulated electron dose on site-specific radiation damage

**Figure 4:**
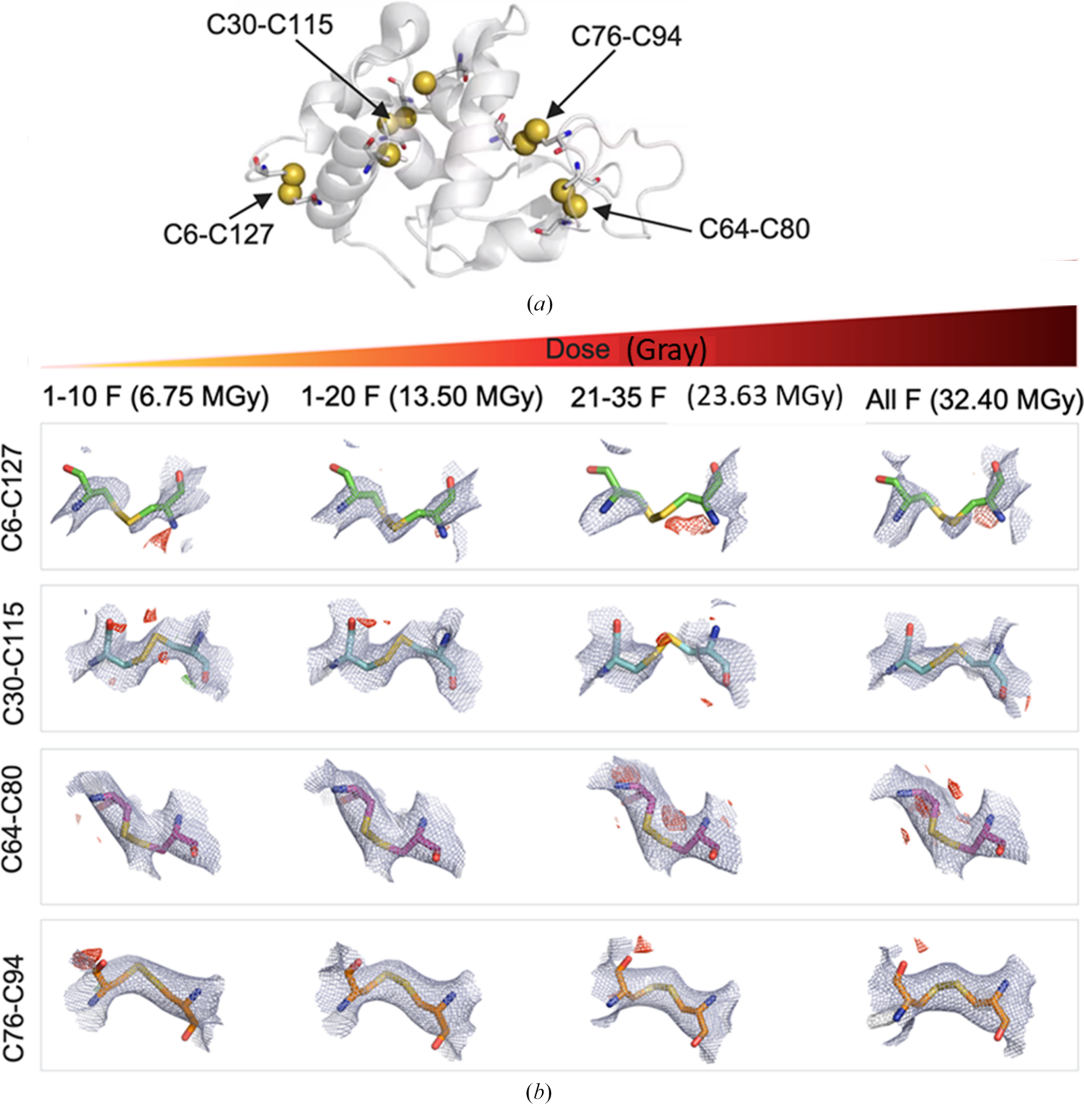
Site-specific damage of disulfide bonds in lysozyme:native as a function of electron dose. (*a*) Positions of the four disulfide bonds in hen egg-white lysozyme. (*b*) The 2*mF*_o_ − *DF*_c_ charge-density maps are shown as blue meshes with a contour level of 1.2σ above the mean. *mF*_o_ − *DF*_c_ difference densities are shown in green and red for positive and negative density, respectively, contoured at 2.5σ above and below the mean. A section covering 2 Å around atoms is shown for all densities. (*PyMOL*, Schrödinger). The electron doses are as specified in the figure.

Site-specific damage at disulfide bonds in crystal structures can be detected even at extremely low electron-dose exposures (Hattne *et al.*, 2018 ▸). Specifically, signs of site-specific damage to disulfide bonds are observed at electron doses as low as 11.25 MGy, as judged from decay of the charge-density map and the appearance of negative charge-density features (Hattne *et al.*, 2018 ▸). Lysozyme offers one of the best models for studying site-specific radiation damage due to the presence of four disulfide bonds formed by Cys6–Cys127, Cys30–Cys115, Cys64–Cys80 and Cys76–Cys94 (Fig. 4 ▸*a*).

With an increase in accumulated dose, one can observe decay of the 2*mF*_o_ − *DF*_c_ charge-density maps contoured at 2.5σ. This decay was much more pronounced around Cys6–Cys127 and Cys30–Cys115 compared with the other disulfide bonds (Fig. 4 ▸*b* and Supplementary Fig. S3). Interestingly, the 1-10F (6.75 MGy) data set and 1-20F (13.5 MGy) data set resulted in similar and most ideal electrostatic potential maps around disulfide bonds. For the 21-35F data set (23.63 MGy), the electrostatic potential maps around the Cys6–Cys127 and Cys30–Cys115 disulfide bonds are mostly absent, which presumably indicates breakage of the bonds due to high radiation damage.

**Figure 5:**
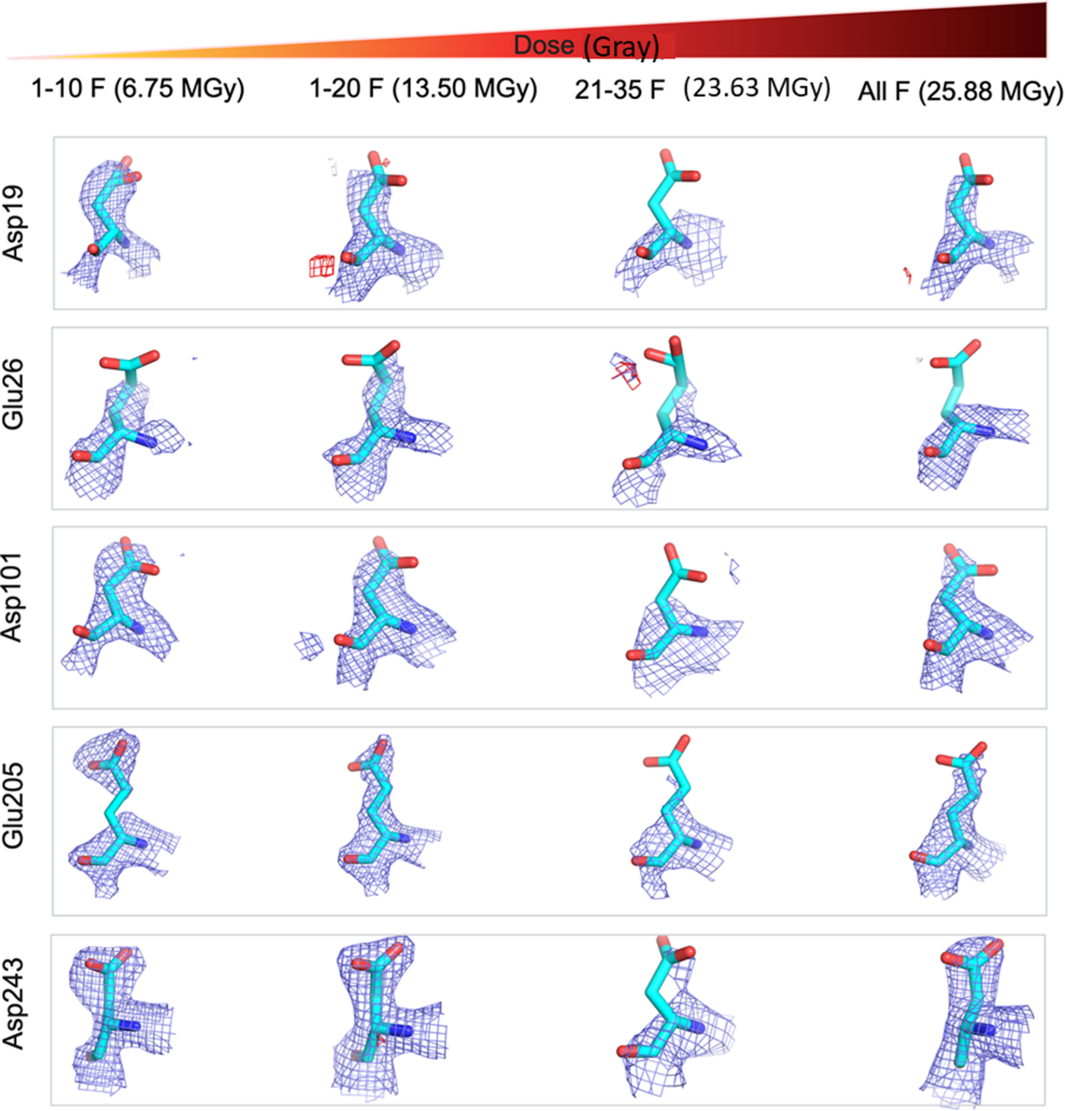
Site-specific damage of aspartic and glutamic acids in the HCA II:AZM protein complex. The 2*mF*_o_ − *DF*_c_ maps are shown as blue meshes with a contour level of 1.5σ above the mean. The *mF*_o_ − *DF*_c_ difference densities are shown in green and red for positive and negative density, respectively, contoured at 3.0σ above and below the mean. A section covering 2 Å around atoms is shown for all densities. The electron doses are as specified in the figure.

Moreover, residues with carboxyl groups in the side chains, such as glutamic acid and aspartic acid, also show signs of site-specific radiation damage through decarboxylation in X-ray crystallography (Weik *et al.*, 2000 ▸) and cryo-EM (Schmid *et al.*, 1992 ▸; Bartesaghi *et al.*, 2014 ▸) as well as in MicroED (Hattne *et al.*, 2018 ▸). The scattering factors for charged O atoms are very low at lower resolutions (Yonekura & Maki-Yonekura, 2016 ▸), so carboxyl density will decrease because of global damage worsening resolution as well as specific damage (Maki-Yonekura *et al.*, 2023 ▸). To further assess the effect of accumulated electron dose on site-specific damage, we compared the density maps around glutamic acid and aspartic acid residues. There was a systematic decay of density around the carboxylic acid moieties with increasing electron dose for the selected Glu and Asp residues in the HCA II:AZM protein complex (Fig. 5 ▸), as well as in lysozyme:native (Supplementary Fig. S4). As expected, this decay was even more pronounced in the 21-35F data sets since this section of the data mostly contains frames with extremely high electron doses.

### 3.5. The effect of increasing electron dose on protein–ligand binding properties

**Figure 6:**
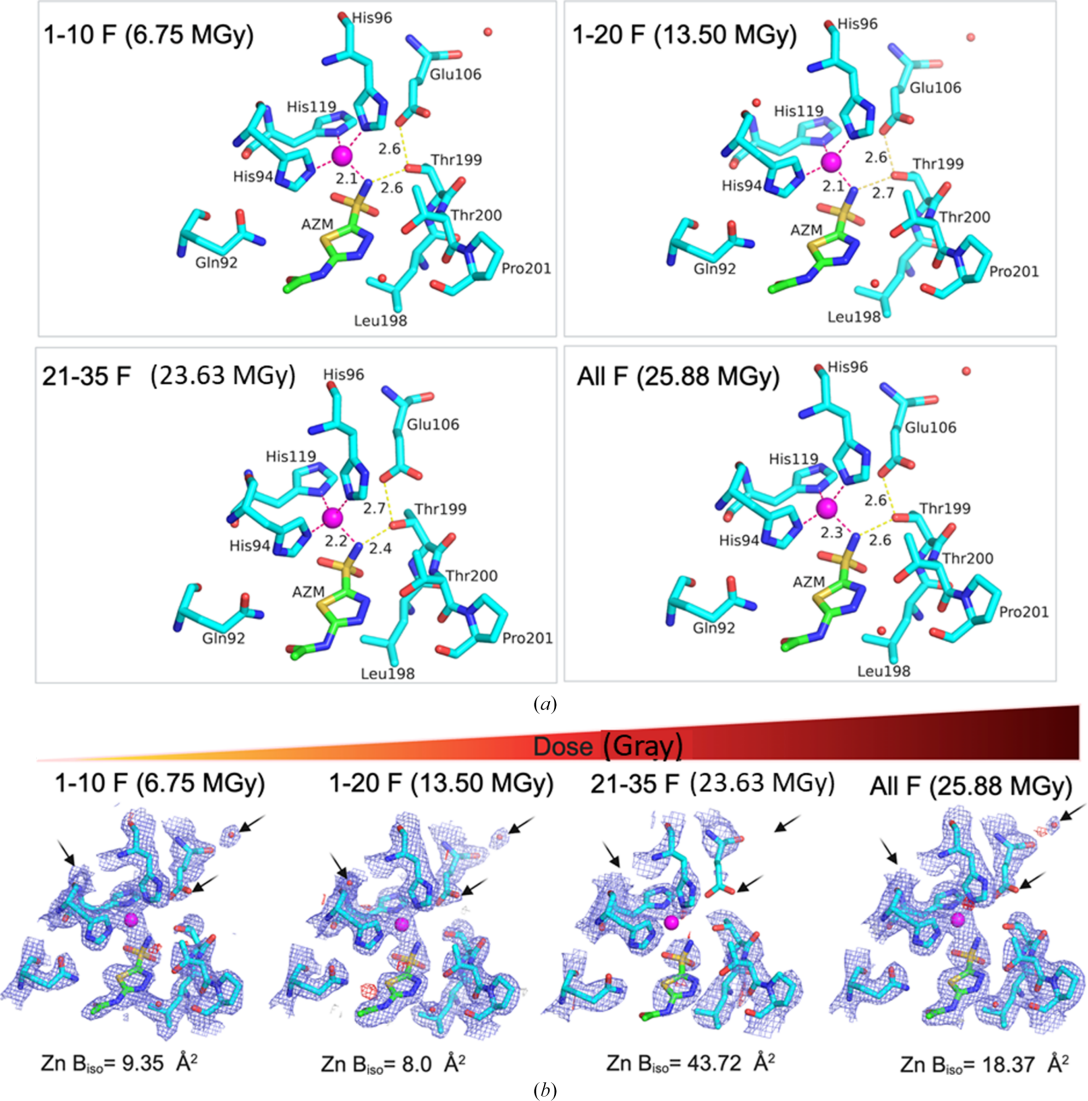
How radiation damage affects the quality of protein–ligand binding properties. (*a*) Polar contacts between HCA II and its inhibitor AZM. (*b*) The quality of the density maps around the HCA II:AZM complex and zinc atomic *B* factors. 2*mF*_o_ − *DF*_c_ maps are shown as blue meshes with a contour level of 1.5σ above the mean. *mF*_o_ − *DF*_c_ difference densities are shown in green and red for positive and negative density, respectively, contoured at 3σ above and below the mean. The 1-10F (6.75 MGy) structure shows some signs of incompleteness, while the 21-35F and All F structures exhibit signs of radiation damage as judged from 2*mF*_o_ − *DF*_c_ maps and increased *mF*_o_ − *DF*_c_ features. The 1-20F data set is more complete with low signs of radiation damage and has the lowest isotropic *B* factor for the active-site zinc metal. The electron doses are as specified in the figure.

To comprehensively study the effect of our data-processing strategy on protein–ligand binding properties, we investigated the ligand-binding interactions between HCA II and its inhibitor AZM (Fig. 6 ▸). As reported previously, binding of AZM to the active site of HCA II is coordinated by the NH of the sulfonamide group of AZM and three active-site histidine residues (His94, His96 and His119), which coordinate the active-site Zn^2+^ ion (Sippel *et al.*, 2009 ▸; Fisher *et al.*, 2012 ▸). This results in a tetrahedral geometry of ligand and residues around the central Zn^2+^ ion.

We also compared the electrostatic potential maps around the ligand, Zn^2+^ ion and the active-site histidine residues. The results reveal a loss of charge density (blue-coloured mesh) with increasing electron dose, some examples of which are highlighted with black arrows (Fig. 6 ▸*b*). Further, as reported in Fig. 6 ▸(*b*), higher electron doses were associated with higher atomic *B* factors of the active-site Zn^2+^ ion; that is, 9.35 and 8.0 Å^2^ for 1-10F and 1-20F, respectively, compared with 43.72 and 18.37 Å^2^ in the case of the 21-35F and All F data sets, respectively.

Moreover, there were more water molecules observed in the 1-20F (13.5 MGy) structures compared with the structures from 21-35F (23.63 MGy) and All F (23.94 MGy). More notably, the numbers of water molecules were 34 in the 1-10F structure and 47 in the 1-20F structure, compared with 31 water molecules in the All F structure (Table 3 ▸). Previous neutron diffraction studies have also shown that at least six water molecules occupy the active site of unbound HCA II, which results in the formation of a network of hydrogen bonds with hydrophilic residues (Fisher *et al.*, 2011 ▸). On binding AZM in the active site, these water molecules are displaced. In Fig. 6 ▸, we observed three of the displaced water molecules in the case of 1-20F compared with two water molecules for the All F data set. These results show that by limiting the accumulated fluence to 3.0 e^−^ Å^−2^ in data processing, it is possible to reveal more structural details in the final crystal structures. Additionally, this data-processing strategy can be adopted to improve the resolution (Table 4 ▸ and Supplementary Tables S2 and S4) and thus the amount of discernible detail in a crystal structure, for example modelling water molecules as well as ligand-binding details such as where hydrogen bonds are likely to form.

### 3.6. The effect of electron dose on radiation damage-induced atomic displacement of metal cations (lysozyme:GdCl_3_)

**Figure 7:**
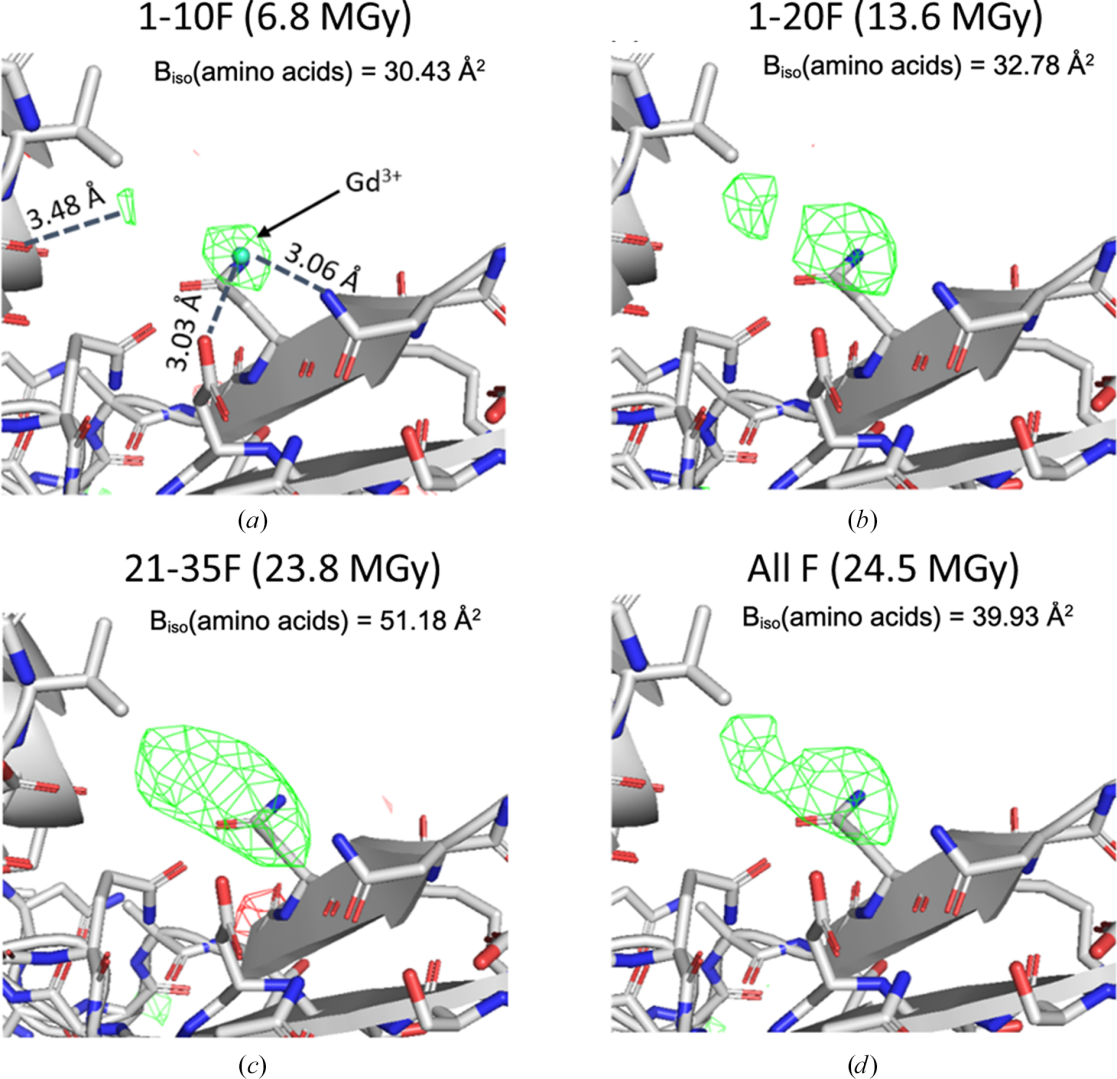
Radiation damage-induced atomic displacement of the soaked metal cation in lysozyme:GdCl_3_. (*a*)–(*d*) The difference charge-density maps (*mF*_o_ − *DF*_c_) of the Gd^3+^ position obtained from refinement against MicroED data with different radiation doses. The positive *mF*_o_ − *DF*_c_ difference densities are shown in green and the negative difference densities are shown in red. All difference density maps are contoured at ±3.5σ. A carve of 2 Å around atoms is assigned for all densities.

In order to study the effect of electron dose on the radiation damage-induced atomic displacement of metal cations, we analysed the difference charge-density maps for the position of Gd^3+^ in tetragonal lysozyme crystals when the electron radiation dose increases. There is one strong difference density peak near amino acids Asp52 and Asn46, as shown in Fig. 7 ▸(*a*), which can be interpreted as Gd^3+^ forming inter­actions with the O and N atoms in the side chains of these two amino acids. There is a second, weaker peak nearby, which could be another Gd^3+^ ion, as discussed in previous studies (Kurachi *et al.*, 1975 ▸; Secemski & Lienhard, 1974 ▸). Similar to the above discussions, the atomic *B* factor of the macromolecule was increased when the dose was increased (Fig. 7 ▸). A quantitative description of the Gd^3+^ atomic displacement (*B* factor) is challenging because of the relatively low data resolution and completeness. However, the shape of the difference charge map for Gd^3+^ was changed from a round and isolated sphere to an elongated blob, as shown in Figs. 7 ▸(*a*), 7 ▸(*b*) and 7 ▸(*d*), indicating an increase in the the mobility of the ion when the electron dose was increased from 6.8 to 13.6 to 24.5 MGy. The difference map obtained from the data set with 13.6 MGy dose is slightly better than that obtained from the data set with 6.8 MGy dose, probably because the change introduced by radiation damage is relatively small, while other effects, such as dynamical effects and data completeness, may contribute to the final map quality. When the 21-35F data set (23.8 MGy) was used, the two charge-density peaks were merged into a blob, making it difficult to locate the Gd^3+^ position. This observation suggests that by processing data with a certain low-dose cutoff, the radiation damage-induced atomic displacement of metal cations can be minimized.

## 4. Discussion

Throughout this work, our results indicate that a low accumulated electron dose of 13.5 MGy, corresponding to a total fluence of 3.0 e^−^ Å^−2^ at 200 kV or 3.6 e^−^ Å^−2^ at 300 kV, was ideal because of (i) the relatively high data completeness, (ii) the high *I*/σ(*I*) and CC_1/2_, (iii) the low *R*_meas_ and (iv) few signs of both global and site-specific radiation damage. This was demonstrated by (i) lower atomic *B* factors, (ii) higher quality density maps and (iii) enhanced detail of the protein–ligand binding interactions. The results support the strategy of using the first few ‘fresh’ frames of diffraction data from a number of randomly oriented crystals found on a cryo-grid when determining the crystal structure, hence omitting later frames that are collected when the crystal is exposed to increasing amounts of radiation.

The global effects of radiation damage in MicroED can be observed at electron doses as low as 4.5 MGy from the decreasing intensity of reflections (Hattne *et al.*, 2018 ▸). While some of the earlier studies proposed appropriate scaling as a measure to compensate for radiation damage (Diederichs, 2006 ▸), such measures need to be complemented with improved data-processing strategies to mitigate radiation damage and to attain higher quality data for macromolecular structure determination. One such strategy, which is well understood and commonly used in X-ray crystallography (Holton, 2009 ▸), is to omit frames that contain data from highly damaged crystals. We note that the critical dose is sample-dependent. It should be estimated based on the inspection of individual electron diffraction patterns in MicroED data sets, data-processing statistics, the refined structure models and electrostatic potential maps.

With recent developments in instrumentation and methods, it is now possible to reduce electron beam damage by collecting near-complete data (completeness > 80%) at low fluence (less than 1 e^−^ Å^−2^) using direct electron detectors in counting mode (Clabbers *et al.*, 2022 ▸; Hattne *et al.*, 2023 ▸). Alternatively, data-collection strategies such as recording many small wedges that together cover a wide overall tilt range (Wang *et al.*, 2025 ▸), or using serial electron diffraction (SerialED), can improve the signal-to-noise ratio while at the same time reducing radiation damage (Smeets *et al.*, 2018 ▸; Bücker *et al.*, 2020 ▸; Hofer *et al.*, 2025 ▸).

We note that the MicroED data for lysozyme:native and HCA II:AZM were collected on a TEM with a LaB_6_ filament, operated at 200 kV, while the MicroED data for lysozyme:GdCl_3_ were collected on a TEM with a field emission gun, operated at 300 kV. For specimens thicker than 100 nm, as is commonly found in MicroED experiments, the radiation damage caused by 300 kV electrons is less than that caused by 200 kV electrons per useful elastic scattering event (Peet *et al.*, 2019 ▸). Therefore, the radiation analysis in the case of lysozyme:GdCl_3_ is not directly comparable with the other samples. From our experimental results, it was shown that the radiation damage caused by 300 kV electrons was less than in the case of 200 kV at the same flux density in e^−^ Å^−2^ s^−1^ and accumulated fluence in e^−^ Å^−2^ (Fig. 2 ▸*b*).

In addition, radiation damage due to accumulated electron dose is dependent on the sample quality and the obtainable resolution as well as the protein identity. Hence, it is seen both from the diffraction-spot intensities of high-dose frames and the final structures that HCA II:AZM crystals are more sensitive to radiation damage compared with lysozyme:native and lysozyme:GdCl_3_ crystals, which makes the application of a one-size-fits-all approach unrealistic. Nevertheless, the absolute critical dose limit certainly varies from sample to sample and is specific for different spatial frequencies, while the trend of electron irradiation-induced damage holds true.

We note that beam damage in MicroED is sample-dependent, as can be seen in our result that the HCA II:AZM crystal is more beam-sensitive compared with the native lysozyme crystals. Therefore, we recommend that the data-collection and processing strategy should be tailored for the specific sample under study, based on visual inspection of the diffraction frames and analyses of data-processing statistics. The proposed approach can be used to obtain diffraction data with low radiation damage and reasonably high completeness, which can result in high-quality structure models.

## 5. Conclusions and outlook

Our results demonstrate that by utilizing only the first few frames of data collected over a large number of randomly oriented crystals, it is possible to improve the recorded ED intensities, the data statistics and the resolution of the final structure. By using the proposed data-processing strategy, we could improve the quality of the data statistics and the resolution of refined crystal structures determined by MicroED. In addition, this strategy could benefit structure-based drug discovery owing to the improved details of protein–ligand binding interaction that can be achieved using the suggested data-processing strategy. The strategy proposed in this study can be applied to a wide array of beam-sensitive samples.

This work builds upon years of method optimization in cryo-EM, X-ray crystallography and previous studies of radiation damage in MicroED, and shows that there is still room for improvement. We encourage the electron crystallo­graphy community to use the correct terminologies when describing electron ‘dose’. Furthermore, automation of data-processing software to extract the best frames with accurate intensities, *I*/σ(*I*) and CC_1/2_ values could benefit crystallo­graphers since it may prove costly in time to manually evaluate data frames given the increasing data collected to date.

## Supplementary Material

Supplementary Figures and Tables. DOI: 10.1107/S205979832500912X/xh5060sup1.pdf

## Figures and Tables

**Table 1:** Terminology used for describing ‘dose’

Term	Qualitative description	Unit (X-rays)	Unit (electrons)
Flux	Particles delivered per unit time	Photons s^−1^	e^−^ s^−1^
Fluence	Particles delivered per unit area	Photons µm^−2^	e^−^ Å^−2^
Flux density	Fluence delivered per unit time	Photons µm^−2^ s^−1^	e^−^ Å^−2^ s^−1^
Dose	Energy absorbed per unit mass	Gray (Gy; J kg^−1^)	Gray (Gy; J kg^−1^)

**Table 2:** Calculated total absorbed doses (MGy) for different data sets and proteins The exposure time (time per frame) is 1.5 s for lysozyme:native and HCA II:AZM and 2 s for lysozyme:GdCl_3_. Calculations are based on the stopping power of H_2_O taken from the ESTAR database (Berger *et al.*, 1999 ▸).

			Total absorbed dose (MGy)			
Sample	Accelerating voltage (kV)	Flux density (e^−^ Å^−2^ s^−1^)	1-10F	1-20F	21-35F	All data
Lysozyme:native	200	0.1	6.75	13.5	23.63	32.4
HCA II:AZM	200	0.1	6.75	13.5	23.63	25.88
Lysozyme:GdCl_3_	300	0.09	6.8	13.6	23.8	24.5

**Table 3:** Data-collection and refinement statistics for lysozyme:native Values in parentheses are for the highest resolution shell. A total of 19 crystals were merged.

Data set	1-10F	1-20F	21-35F	All data
Data collection				
Space group	*P*4_3_2_1_2	*P*4_3_2_1_2	*P*4_3_2_1_2	*P*4_3_2_1_2
Accelerating voltage (kV)	200	200	200	200
*a*, *b*, *c* (Å)	77.32, 77.32, 38.50	77.45, 77.45, 38.25	77.51, 77.51, 38.12	77.46, 77.46, 38.22
α, β, γ (°)	90, 90, 90	90, 90, 90	90, 90, 90	90, 90, 90
Resolution (Å)	27.34–3.00 (3.11–3.00)	27.21–3.00 (3.11–3.00)	25.65–3.00 (3.11–3.00)	27.39–3.00 (3.11–3.00)
Multiplicity	14.8 (15.0)	25.5 (26.2)	15.6 (16.5)	45.5 (49.0)
Completeness (%)	63.21 (65.84)	71.35 (71.54)	71.67 (62.40)	76.71 (76.68)
*R*_meas_	0.565 (2.695)	0.687 (5.063)	1.038 (16.61)	0.844 (8.263)
*R*_p.i.m._	0.141 (0.641)	0.129 (0.900)	0.2423 (3.721)	0.1152 (1.056)
〈*I*/σ(*I*)〉	4.78 (1.90)	5.33 (1.93)	2.74 (0.73)	5.81 (1.92)
CC_1/2_	0.970 (0.645)	0.958 (0.548)	0.888 (0.164)	0.969 (0.300)
Wilson *B* factor (Å^2^)	66.30	68.83	78.80	72.78
Refinement				
Total reflections	24043 (2406)	46964 (4952)	30565 (3187)	89810 (9560)
Unique reflections	1627 (160)	1844 (189)	1956 (193)	1976 (195)
*R*_work_	0.2217 (0.3444)	0.2194 (0.3318)	0.2706 (0.3862)	0.2376 (0.3584)
*R*_free_	0.2749 (0.3501)	0.2376 (0.3532)	0.3142 (0.4563)	0.2786 (0.4205)
No. of atoms				
Total	1014	1014	1014	1014
Macromolecules	1012	1012	1012	1012
Ligands	2	2	2	2
Protein residues	129	129	129	129
R.m.s. deviations				
Bond lengths (Å)	0.003	0.003	0.003	0.003
Bond angles (°)	0.53	0.52	0.57	0.53
Ramachandran statistics				
Favoured (%)	97.64	98.43	98.43	98.43
Allowed (%)	2.36	1.57	1.57	1.57
Outliers (%)	0.00	0.00	0.00	0.00
Rotamer outliers (%)	1.83	1.83	0.92	0.92
Clashscore	2.51	2.51	3.01	2.51
Average *B* factors (Å^2^)				
Overall	56.06	58.13	67.58	62.78
Macromolecules	56.05	58.12	67.58	62.78
Ligands	60.20	60.75	68.94	64.12

**Table 4:** Data-collection and refinement statistics for HCA II:AZM Values in parentheses are for the highest resolution shell. A total of 20 crystals were merged.

Data set	1-10F	1-20F	21-35F	All data
Data collection				
Space group	*P*2_1_	*P*2_1_	*P*2_1_	*P*2_1_
*a*, *b*, *c* (Å)	42.30, 41.79, 72.92	42.33, 41.76, 72.82	42.40, 41.84, 72.75	42.35, 41.75, 72.92
α, β, γ (°)	90, 102.885, 90	90, 102.742, 90	90, 102.848, 90	90, 102.819, 90
Resolution (Å)	28.79–2.25 (2.33–2.25)	27.05–2.25 (2.33–2.25)	28.83–2.80 (2.90–2.80)	28.79–2.25 (2.33–2.25)
Multiplicity	3.0 (2.1)	4.7 (3.0)	3.7 (2.9)	6.7 (4.1)
Completeness (%)	67.60 (60.68)	84.85 (77.06)	73.22 (58.51)	94.17 (86.06)
*R*_meas_	0.352 (1.215)	0.486 (1.804)	0.590 (3.173)	0.579 (2.637)
*R*_p.i.m._	0.173 (0.701)	0.190 (0.895)	0.281 (1.669)	0.191 (1.150)
〈*I*/σ(*I*)〉	2.95 (1.08)	3.27 (1.04)	2.64 (0.62)	3.12 (0.83)
CC_1/2_	0.94 (0.347)	0.94 (0.237)	0.892 (0.250)	0.926 (0.105)
Wilson *B* factor (Å^2^)	19.65	22.68	46.94	29.73
Refinement				
Total reflections	24513 (1500)	48167 (2825)	17358 (1367)	75970 (4381)
Unique reflections	8128 (730)	10217 (927)	4743 (471)	11413 (1071)
*R*_work_	0.2275 (0.3083)	0.2311 (0.3428)	0.2527 (0.3433)	0.2318 (0.3441)
*R*_free_	0.2728 (0.3239)	0.2607 (0.3075)	0.3159 (0.2884)	0.2768 (0.3831)
No. of atoms				
Non-H	2101	2114	2067	2098
Macromolecules	2049	2049	2049	2049
Ligand/ion	18	18	18	18
Solvent	34	47	0	31
Protein residues	257	257	257	257
Average *B* factors (Å^2^)				
Overall	18.39	21.07	33.47	30.46
Macromolecules	18.51	21.16	33.39	30.58
Ligand/ion	15.87	22.05	42.31	32.37
Solvent	12.44	16.60	_	30.00
R.m.s. deviations				
Bond lengths (Å)	0.003	0.003	0.003	0.003
Bond angles (°)	0.79	0.80	0.75	0.74
Ramachandran statistics				
Favoured (%)	94.90	96.86	95.29	95.29
Allowed (%)	5.10	3.14	4.71	4.71
Outliers (%)	0.00	0.00	0.00	0.00
Rotamer outliers (%)	0.0	0.00	0.00	0.00
Clashscore	5.46	5.15	4.66	6.13

**Table 5:** Data-collection and refinement statistics for lysozyme:GdCl_3_ Values in parentheses are for the highest resolution shell. A total of nine crystals were merged.

Data set	1-10F	1-20F	21-35F	All data
Data collection				
Space group	*P*4_3_2_1_2	*P*4_3_2_1_2	*P*4_3_2_1_2	*P*4_3_2_1_2
Accelerating voltage (kV)	300	300	300	300
*a*, *b*, *c* (Å)	80.13, 80.13, 36.95	80.24, 80.24, 37.16	80.21, 80.21, 38.09	80.15, 80.15, 37.49
α, β, γ (°)	90, 90, 90	90, 90, 90	90, 90, 90	90, 90, 90
Resolution (Å)	35.84–3.00 (3.11–3.00)	35.89–3.00 (3.11–3.00)	35.87–3.00 (3.11–3.00)	35.85–3.00 (3.11–3.00)
*R*_meas_	0.511 (1.720)	0.438 (1.671)	0.535 (3.744)	0.501 (1.968)
*R*_p.i.m._	0.153 (0.513)	0.103 (0.369)	0.157 (0.721)	0.096 (0.377)
〈*I*/σ(*I*)〉	4.3 (1.6)	6.0 (2.2)	4.6 (1.0)	6.1 (2.3)
CC_1/2_ (%)	91.5 (54.4)	94.3 (79.8)	93.9 (4.8)	92.1 (53.9)
Completeness (%)	81.9 (84.0)	90.5 (88.4)	73.6 (74.0)	93.6 (90.9)
Wilson *B* factor (Å^2^)	36.25	41.31	60.08	46.83
Refinement				
No. of reflections	2173	2420	2011	2520
No. of unique reflections	221	237	191	240
*R*_work_	0.2521 (0.2806)	0.2736 (0.2900)	0.2701 (0.2936)	0.2883 (0.2990)
*R*_free_	0.3250 (0.3465)	0.3385 (0.4251)	0.3464 (0.5797)	0.3484 (0.4277)
No. of atoms				
Non-H	992	992	992	992
*B* factor (Å^2^)				
Protein	30.43	32.78	51.18	39.93
R.m.s. deviations				
Bond lengths (Å)	0.010	0.003	0.008	0.003
Bond angles (°)	1.140	0.787	1.100	0.792
Ramachandran statistics				
Favoured (%)	98.43	98.43	98.43	98.43
Allowed (%)	1.57	1.57	1.57	1.57
Outliers (%)	0.00	0.00	0.00	0.00
Rotamer outliers (%)	0.00	0.00	0.00	0.00
Clashscore	2.55	4.08	3.60	4.10
