# Supplementary material for: Limiting the effects of radiation damage in MicroED through dose selection during data processing
Source: Acta Crystallogr D Struct Biol. 2025 Nov 13;81(Pt 12):693–707. doi: 10.1107/S205979832500912X (PMC12809500; doi:10.1107/S205979832500912X)
Supplement: Supplementary file 1 [file d-81-00693-sup1.pdf]

STRUCTURAL
BIOLOGY

Volume 81 (2025)

Supporting information for article:

Limiting the effects of radiation damage in MicroED through dose selection during data processing

Hongyi Xu, Henri Colyn Bwanika, Jingjing Zhao, Gerhard Hofer, Uwe H. Sauer and Hongyi Xu

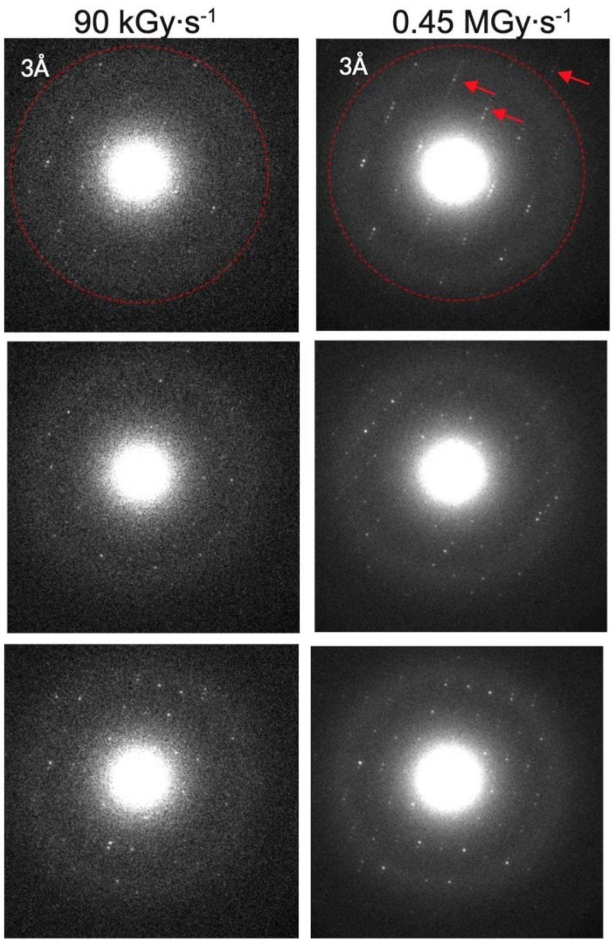

Figure S1 Comparison of electron diffraction patterns collected at lower flux density / dose rate (0.02 $e^{-}\cdot Å^{-2}\cdot s^{-1}$ / 90 $kGy\cdot s^{-1}$) and high flux density such as the one used in this study (0.1 $e^{-}\cdot Å^{-2}\cdot s^{-1}$ / 0.45 $MGy\cdot s^{-1}$) at 200 kV. We note that data collected at 0.1 $e^{-}\cdot Å^{-2}\cdot s^{-1}$ has better signal-to-noise ratio. More diffraction spots are visible at 0.1 $e^{-}\cdot Å^{-2}\cdot s^{-1}$.

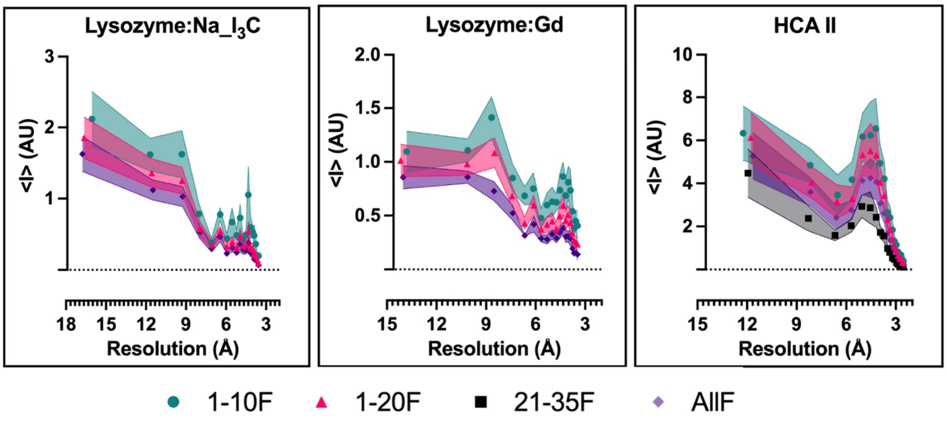

Figure S2 Effect of electron dose on the strength of integrated and unmerged mean reflection intensities <I> based on Wilson statistics**.** Intensity of diffraction spots as a function of resolution. 1-10F- First ten frames dataset, 1-20F- First twenty frames dataset, 21-35F -frame range between twenty one to thirthy five. All F- all data. Overall, the 1-10F dataset yielded reflections with higher mean intensities compared to other datasets. Data are plotted as <I> = Mean I_{hkl} ±Standard Error of the Mean versus resolution, representative 15 crystal (lysozyme:Na_I_3C), 13 crystals (HCA II) and 14 crystals (Lysozyme:Gd). Note that in each case the same crystals are compared for the different doses. Lysozyme:Na_1_3C refers to lysozyme bound to $C_8H_4I_3NO_4$ molecules, lysozyme:Gd refers to lysozyme soaked in $GdCl_3$. The flux density is 0.1 $e^{-}\cdot Å^{-2}\cdot s^{-1}$, the exposure time is 1.5 s per frame for Lysozyme:Na_I_3C and HCA II and 2 s per frame for Lysozyme:Gd.

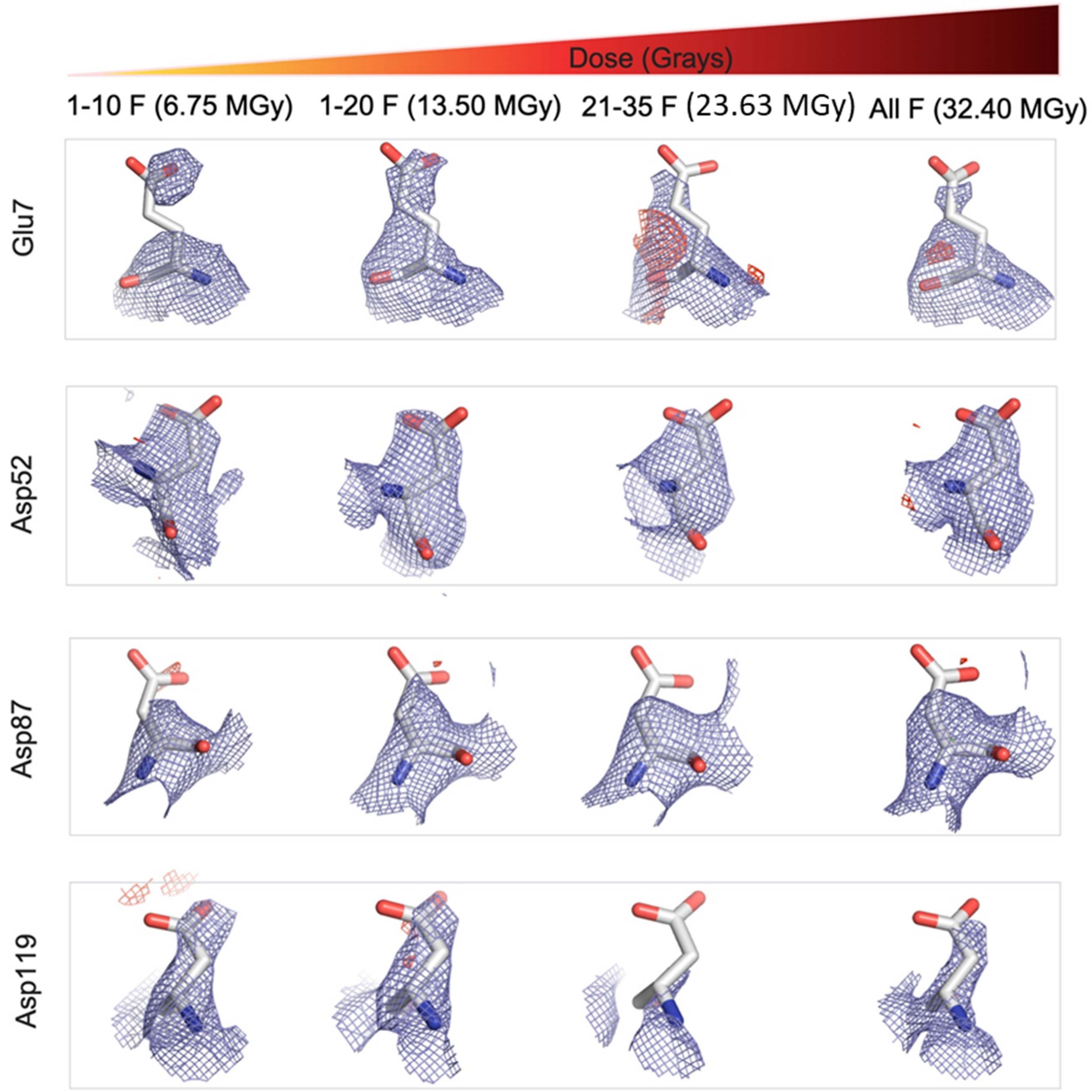

Figure S3 Site specific damage of acidic moities in aspartic acid and glutamic acid in Lysozyme:native protein. The $2m\mathrm{F_o}{-}D\mathrm{F_c}$ maps are shown in blue meshes with a contour level of 1.2σ above the mean. The $m\mathrm{F_o}{-}D\mathrm{F_c}$ difference densities are shown in green and red for positive and negative density respectively, contoured at 2.5σ above and below the mean. A section covering 2 Å around atoms is shown for all densities. The electron doses are as specified in the figure.

Table S1 Average unit cell parameters of merged lysozyme:native crystals for respective frame number data sets generated from XDS (Kabsch 2010b).

Data set (Frames)	Unit Cell Parameters		
	a = *b* (Å)	*c* (Å)	*α* = *β* = *γ* (°)
**1-10 F**	77.32	38.50	90
**1-20 F**	77.45	38.25	90
**21-35 F**	77.51	38.12	90
**All F**	77.47	38.22	90

Table S2 Overall data merging statistics of lysozyme:native crystals for respective frame number data sets generated from XDS (Kabsch 2010b).

Resolution range	Observed reflections	Unique reflections	Completeness (%)	R-meas	I/σ(I)	$CC_{1/2}$ (%)
12.0 – 3.0	24043	1627	63.2	56.5	4.78	97.0*
12.73 – 3.0	46969	1845	71.8	68.4	5.33	96.2*
13.42 – 3.0	30570	1957	76.4	103.1	2.75	90.0*
13.42 – 3.0	89867	1977	77.0	84.4	5.81	96.8*

Order of data sets as in **Supplementary Table S1**

Table S3 Average unit cell parameters of merged HCA II:AZM crystals for respective frame number data sets generated from XDS (Kabsch 2010b).

Data set (Frames)	Unit Cell Parameters					
	a (Å)	*b* (Å)	*c* (Å)	*α* (°)	*β* (°)	*γ* (°)
**1-10 F**	42.296	41.792	72.923	90	102.885	90
**1-20 F**	42.329	41.761	72.823	90	102.742	90
**21-35 F**	42.402	41.843	72.745	90	102.848	90
**All F**	42.35	41.754	72.92	90	102.819	90

Table S4 Overall data merging statistics of HCA II:AZM crystals for respective frame number data sets generated from XDS (Kabsch 2010b).

Resolution range	Observed reflections	Unique reflections	Completeness (%)	R-meas (%)	I/σ(I)	$CC_{1/2}$ (%)
10.06 – 2.25	24515	8129	67.6	35.2	2.95	93.8*
10.06 – 2.25	48174	10218	85.0	48.6	3.27	93.9*
10.06 – 2.25	26329	8780	72.9	75.0	1.55	86.6*
10.06 – 2.25	11415	94.8	57.9	3.12	93.4*	75983

Order of data sets as in **Supplementary Table S3**

Table S5 Average unit cell parameters of merged Lysozyme:$GdCl_3$ crystals for respective frame number data sets generated from XDS (Kabsch 2010b).

Data set (Frames)	Unit Cell Parameters		
	$a = b$ (Å)	c (Å)	$\alpha = \beta = \gamma$ (°)
**1-10 F**	80.13	36.95	90
**1-20 F**	80.24	37.17	90
**21-35 F**	80.24	38.09	90
**All F**	80.16	37.49	90
